# Obituary for Herman D. Suit, M.D., D. Phil.

**DOI:** 10.1007/s00066-022-02004-2

**Published:** 2022-09-30

**Authors:** Michael Baumann, Wilfried Budach, Jürgen Debus

**Affiliations:** 1 Deutsches Krebsforschungszentrum, Im Neuenheimer Feld 280, 69120 Heidelberg, Germany; 2 Klinik für Strahlentherapie und Onkologie, Universitätsklinikum Düsseldorf, Düsseldorf, Germany; 3 Klinik für Radioonkologie und Strahlentherapie, Universitätsklinikum Heidelberg, Heidelberg, Germany

**Fig. 1:**
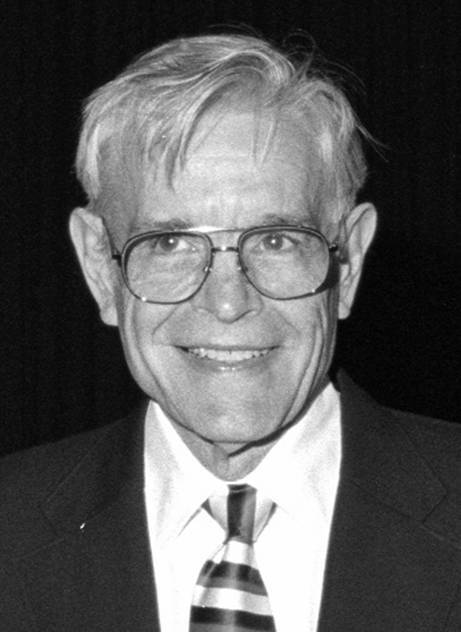
Herman D. Suit | ©Photo: German Röntgen Museum, Remscheid (with kind permission to publish in the current article)

Herman D. Suit, M.D., D. Phil. (Fig. 1), was among the most important radiation oncologists shaping our discipline, and an honorary member of the *Deutsche Gesellschaft für Radioonkologie* (DEGRO). In July 2022, Dr. Suit passed away at the age of 93.

Herman Suit, born 1929 in Waco Texas, began his medical studies at Baylor Medical School in Houston at the age of 19 [2]. A summer course at the University of Texas then sparked Suit’s enthusiasm for radiation therapy, which would remain with him throughout his life [2]. He earned his D. Phil. in Radiation Oncology at the University of Oxford, where he studied the effect of radiation on the cellularity of human bone marrow, while simultaneously devoting himself to clinical oncology under another pioneer of radiotherapy, Dr. Frank Ellis [3].

Back in the USA, he initially went to the National Cancer Institute in Bethesda, Maryland, before returning to his origins in Texas in 1959. At MD Anderson Cancer Center Houston, under mentorship of Gilbert Fletcher, at that time* the* leader in international radiotherapy, he started to establish the principles in the treatment of soft tissue sarcomas by radiation and conservative surgery rather than amputation. In Houston, he also developed the Fletcher–Suit applicator for intracavitary radiation for women with cervical and endometrial cancer and, moreover, established one of the most important mouse colonies in the world [3].

In 1970, Herman Suit joined Harvard Medical School as Professor of Radiation Oncology, and in 1971, he also became Head of Radiation Medicine at Massachusetts General Hospital, where he later held the Andres Soriano Professorship of Radiation Oncology [4].

Hermans Suit’s lifetime achievements cannot be overstated. Always a strict advocate of scientific rigor (“you are not here to prove your idea, you are here to thoroughly investigate it”) and evidence (“what is the data?”), he was a visionary striving to move things forward. His laboratory work was vastly important in understanding the mechanisms governing the radiation response of tumors and normal tissues. Dr. Suit used his wide radiobiological and clinical knowledge to advance the safe and successful use of radiation therapy, whether through judicious fractionation, radiation doses based on tumor volume and type, shrinking fields, or the integration of radiation with surgery or chemotherapy. He also was an advocate of pushing anatomical precision of radiotherapy to its limits, not least to keep the risk of secondary tumor induction by radiotherapy as low as possible. In this context, he was one of the pioneers of making proton beam therapy a clinical reality [2, 3]. However, his most widely known contribution to the general field oncology will remain his seminal work ensuring that soft tissue sarcomas, which could previously only be radically operated on, are today equally or even more successfully treated with combination therapies—with limbs being spared [3]. Dr. Suit lifelong passion for science was fueled by his wish to make therapies better for patients. This patient-centered ethos and empathic personality was experienced by anybody who worked with him in clinics or designed research programs for which Dr. Suit regularly queried the potential impact for clinical care.

Dr. Suit was also very active in serving our community in leadership positions, for example as president of the Radiation Research Society or the American Society of Therapeutic Radiation Oncology (ASTRO). Herman Suit’s achievements were recognized, for example, with the Gold Medal of ASTRO and the American College of Radiology, and the Charles Kettering Award from the General Motors Cancer Research Foundation. In Germany, he received the Roentgen Medal in 2001 and was made an Honorary Member of DEGRO in 2002 [5, 6].

During his time at MGH, Dr. Suit hosted many young talents from all over the world. National borders played no role for him, he recruited the brightest minds wherever he could find them. He was a devoted and outstanding mentor, a role model in scientific integrity and ambition, and a life-long fatherly friend, following with great interest the careers of his former trainees of whom many later became leading international radiation oncologists and radiation researchers [3].

His special love, however, was his wife Joan Suit, also a fervent scientist and pioneer in the field of bacterial genetics. The two shared a passion for theater and music, which they always felt obliged to pass on to others, for example by regularly giving front row theater and ballet tickets to their trainees. And the Suits were always extremely generous in other ways, too, for example by inviting trainees from abroad and their families for social events at their home (particularly the famous Thanksgiving parties) and contributing to the establishment of the Herman and Joan Suit Professorship in Radiation Oncology at the Massachusetts General Cancer Center [3, 7]. Joan Suit died in 2021 at the age of 90. Now Herman Suit has succeeded her. We will keep him in our memories as a wonderful clinician and scientist, and friend.

*Michael Baumann, Heidelberg*

*Wilfried Budach, Düsseldorf*

*Jürgen Debus, Heidelberg*
